# 

**Published:** 1966-07

**Authors:** 


					1. The Volume number, date, and issue number
should read :?
Vol. 81 (iii) July 1966 No. 301
THE
BRISTOL MEDICO-CHIRURGICAL JOURNAL
(.Incorporating the Medical Journal of the South-West)
Established 18 8 3
Sul
vol. 80 (iii) July 1965 No. 29?
PUBLISHED QUARTERLY
ANNUAL SUBSCRIPTION 16/- POST FREE
p. 37-4^ Duf *6
S<"& zj it rJ-f)C
BRISTOL MEDICO-CHIRURGICAL JOURNAL
PUBLISHED UNDER THE AUSPICES OF THE
Bristol Medico-Chirurgical Society
The Society was founded in 1874. In 1883 publication of this Journal bega'
and has continued quarterly since then. During the years 1949 to 1962
appeared under the title of "The Medical Journal of the South West".
The Society founded a medical library in 1890, which in 1892 was combine
with that of the Bristol Medical School. This became the University of Brist
Medical Library in 1925, and an agreement in that year confirmed tl1
privilege of Members to use the University Medical Library.
Editorial Committee
Dr. N. J. Brown, Southmead Hospital, Bristol. Joint Editor.
Dr. A. B. Raper, Department of Pathology, Bristol Royal Infirmary.
Joint Edito1
Dr. J. Macrae, Ham Green Hospital.
Dr. R. C. Wofinden, Central Clinic, Bristol.
Mr. A. E. S. Roberts, Medical Library, University of Bristol. Secretary.
Bath
Exeter .
Gloucester
Plymouth
Taunton
Torquay
Truro .
Yeovil .
Local Correspondents
Mr. A. D. Bateman, 3 The Circus, Bath.
Dr. L. N. Jackson, Mount Jocelyn, Crediton, Devon.
Dr. R. F. Jarrett, 9 College Green, Gloucester.
Dr. C. R. Croft, Lexden, Hartley Av., Plymouth.
Dr. M. McAlley, 1 The Crescent, Taunton.
Dr. A. Robinson Thomas, 20 Devon Square, Newton Abbo1
Devon.
Dr. F. D. M. Hocking, St. Andrews, Perranporth, Cornwall-
Mr. J. A. Vere Nicoll, Knapp House, Yeovil, Somerset.
NOTICE TO CONTRIBUTORS
Original articles are invited, provided they have not been published elsewhere. Not?'
of forthcoming events, meetings held, degrees conferred, staff appointments, and othe'
local news are welcomed. The editorial committee reserves the right to make literal
corrections.
Illustrations should always be supplied ready for reproduction. All re-touchings ?(
other operations required will be charged to the author. Line drawings should be dra^/1
in Indian ink. We regret that we must ask authors to pay for making blocks, if this ?'
necessary.
NOTES AND NEWS
? The following have been elected Fellows of the Royal College of Physicians of
?ndon:
W. Barritt, McD. G. Stewart.
Examination Result
^?R.C.P. M. E. Horn.
1
F. Bailey & Son, Durslcy. u
t, -i

				

